# Long-Term Outcomes and Risk Factors of Mortality After Reoperation on the Aortic Root: A Single-Center 20-Year Experience

**DOI:** 10.3390/jcm14113727

**Published:** 2025-05-26

**Authors:** Nikoleta Bozini, Nicole Piber, Keti Vitanova, Konstantinos Sideris, Ulf Herold, Ralf Guenzinger, Teodora Georgescu, Andrea Amabile, Markus Krane, Anatol Prinzing

**Affiliations:** 1 Department of Cardiovascular Surgery, Institute Insure, German Heart Center Munich, School of Medicine & Health, Technical University of Munich, 80636 Munich, Germany; bozini@dhm.mhn.de (N.B.); piber@dhm.mhn.de (N.P.); vitanova@dhm.mhn.de (K.V.); sideris@dhm.mhn.de (K.S.); herold@dhm.mhn.de (U.H.); guenzinger@dhm.mhn.de (R.G.); georgescu@dhm.mhn.de (T.G.); amabile.andrea@gmail.com (A.A.); 2 Division of Cardiac Surgery, Department of Cardiothoracic Surgery, University of Pittsburgh, Pittsburgh, PA 15213, USA; 3 UPMC Heart and Vascular Institute, University of Pittsburgh Medical Center, Pittsburgh, PA 15213, USA; 4 DZHK (German Center for Cardiovascular Research), Partner Site Munich Heart Alliance, 80636 Munich, Germany; 5 Department of Cardiovascular Surgery, University Hospital and Goethe University Frankfurt, 60323 Frankfurt, Germany; anatol.prinzing@unimedizin-ffm.de

**Keywords:** aortic surgery, aortic valve, aortic root, redo, reoperation, Bentall, follow-up, mortality, outcomes

## Abstract

**Objective:** Over the last ten years, aortic surgery has transitioned from a high-risk procedure to a well-established operation, offering favorable outcomes and survival when performed by experienced hands. Advances in surgical techniques and evolving technologies allow treatment of older and more complex patients with reoperations. However, outcome data are limited. This study aims to identify risk factors for adverse outcomes after reoperation on the aortic root. **Methods**: This retrospective study included patients who received aortic root reoperation from 1999 to 2023 in a high-volume center, with a history of previous surgery on the thoracic aorta or aortic valve. Patients under the age of 18 or those with transcatheter aortic valve implantation as an index procedure were excluded. **Results**: A total of 192 patients were analyzed. Mean age was 57 ± 13 years, and 77.6% were men. The main procedure was Bentall (88.5%). An elective operation was performed in 54.7% of the patients. The mean time between the index operation and reoperation was 8.61 (3.01–16.05) years. Mortality at 30 days was 13%. Survival rates at one, five, and ten years were 84%, 81%, and 71%, respectively. Female gender, non-elective surgery, concomitant procedures, and combined procedures on the aortic root and arch were associated with worse survival. In the Cox regression, age (HR = 3.98, *p* < 0.01), EuroSCORE II (HR = 1.46, *p* < 0.01), concomitant procedures at reoperation (HR = 2.53, *p* = 0.01), prolonged cardiopulmonary bypass time (HR = 1.01, *p* < 0.01), bleeding complications (HR = 6.11, *p* < 0.01), and need for temporary mechanical circulatory support (HR = 4.86, *p* = 0.01) were significantly associated with a higher mortality. Analysis of the receiver operating characteristic curve revealed that age > 60 years at reoperation is a strong predictor for poor outcomes (AUC = 0.712, *p* < 0.01). **Conclusions:** Mortality following aortic root reoperation is primarily driven by baseline patient risk and perioperative complications. Reduced survival was observed in patients over 60 years of age, females, those having non-elective surgery, combined root and arch operations, and procedures with additional concomitant operations. Bleeding events, the use of temporary mechanical circulatory support, and concomitant interventions at reoperation emerged as independent predictors of mortality.

## 1. Introduction

Over the last ten years, aortic surgery has transitioned into a standardized procedure with good outcomes and improved survival. Evolving surgical techniques and technologies have extended patient survival, leading to a rise in the frequency of reoperations [1]. Biological prostheses as aortic valve replacements lead to increasing rates of reoperations involving the aortic root [2]. The most common indications for reoperation are aortic root dilation, structural valve degeneration, aortic dissection, and infective endocarditis [3,4]. Up to date, reoperation on the aortic root is still considered a high-risk intervention with lower overall survival than the initial operation and with a hospital mortality up to 17% [5]. Challenges arise due to the proximity of the ascending aorta to the sternum with possible adhesion after the initial operation, but also the remobilization of the coronary arteries, especially with prior homograft implantation [6]. In previous studies, risk factors, such as cardiopulmonary bypass (CPB) time, endocarditis, and electiveness of the operation, were identified as predictors for adverse outcomes after reoperation [7,8]. Unfortunately, data on reoperations are oftentimes sparse, and the patient population included in these studies is very heterogeneous.

The goal of this study was to demonstrate the long-term outcome after reoperations on the aortic root at our institution over the course of 20 years and to determine predictors of unfavorable outcomes to ensure that reoperation on the aortic root can become a safe and standardized procedure in the future.

## 2. Materials and Methods

### 2.1. Patients and Data Collection

We reviewed all patients undergoing reoperation of the aortic root with or without a replacement of the aortic valve at our institution between 1999 and 2023. We selected patients with a previous operation on the aorta, the aortic valve, or a combination of both. Criteria for exclusion were age under 18 at reoperation or transcatheter aortic valve replacement at the initial procedure. This yielded 192 patients, who were then included in this study. The principal outcome was mortality. Additional outcomes included cardiac re-reoperation, bleeding requiring intervention or operation, new aortic dissection, infective endocarditis, readmission due to cardiac cause, cardiovascular and cerebral complications, permanent pacemaker implantation, and the need for temporary mechanical circulatory support (tMCS). Data were acquired by retrospective review of patient reports, operation reports, telephone interviews with the patients, and follow-up with the primary care physician.

### 2.2. Operative Techniques

At our center, reoperations are routinely performed by experienced senior surgeons to ensure the best possible outcomes. Detailed preoperative imaging, particularly CT scans, is crucial for planning the best reoperation strategy. Standard techniques for aortic root replacement, mostly involving the Bentall procedure, are performed through a median sternotomy. Adjustments are made in certain cases due to the complex pathophysiology of the patients. To minimize exposure to cardiopulmonary bypass, our standard aortic cannulation method after redo sternotomy is central cannulation. Peripheral cannulation and CPB initiated before sternotomy are used only when imaging shows significant proximity of the heart to the sternum. Due to the longer duration of reoperations, we used systemic hypothermia (32 °C) for patients with isolated aortic root replacement and cared for optimal myocardial protection. However, in cases where a concomitant aortic arch replacement—with or without extension to the descending aorta—was performed, systemic cooling to lower temperatures (down to 20 °C) and circulatory arrest via antegrade cerebral perfusion were employed as part of our cerebral protection strategy. Hemodynamic support strategies included the use of inotropes and mechanical circulatory support when indicated, particularly in patients with longer operative times. Routine pulmonary function measurements were not collected as part of this retrospective analysis, while prolonged ventilation time and ICU stay were recorded and analyzed. Mobilization and patency of the coronary arteries are critical in reoperations at the aortic root and play a key role in good outcomes. Therefore, we handle the coronary vessels with utmost caution, paying close attention to the appropriate length to avoid torsion and kinking. Once the proximal and coronary anastomoses are completed, we apply surgical glue to secure the anastomoses, as controlling postoperative bleeding in this area is challenging due to limited access. In younger patients, mechanical valves were typically implanted as the standard approach, except in cases of endocarditis or patients with extensive debridement, where biological prostheses were primarily used.

### 2.3. Statistical Analysis

Categorical variables were summarized as counts and percentages. Continuous variables were described either as medians with interquartile ranges (IQR) or as means with standard deviations. Kaplan–Meier analysis was utilized to estimate overall survival following reoperation. Survival outcomes were compared between subgroups based on the number of electiveness of reoperation, receipt of concomitant procedures, gender, and combined procedures at the aortic arch using the log-rank test. A sensitivity analysis of overall mortality was additionally conducted after stratification for these variables after excluding patients who had the index operation in the previous 30 days. Median survival times and survival probabilities at predefined time points were reported where applicable. Cox proportional hazards models were employed for further survival analyses. Initially, univariate Cox regression analyses were conducted to screen for potential mortality predictors. Variables showing a *p*-value less than 0.10 in the univariate analysis were considered for inclusion in multivariate models. To reduce the risk of overfitting, particularly due to the limited number of events, variables exhibiting strong clinical correlation with other predictors were excluded. Nested multivariate Cox models were constructed, with gender and age included in all analyses. In the final multivariate model, all variables significant in the univariate analysis were tested. Results were presented as hazard ratios (HR) along with 95% confidence intervals (CI). Additionally, receiver operating characteristic (ROC) curve analysis was performed to assess a cut-off age of prognostic significance. Statistical significance was set at a *p*-value < 0.05. All statistical analyses were conducted using SPSS Version 26 (IBM Corp., Armonk, NY, USA) and R Version 4.4.3 (R Core Team, 2024).

## 3. Results

### 3.1. Patient Demographics

A total of 192 patients were included in this study. Patient characteristics are shown in Table 1.

The mean age was 57 ± 13 years, and 77.6% (149/192) of the study population were men. The mean EuroSCORE II was 5.08 (2.38–10.09).

### 3.2. Data on Initial Operation

At the initial operation, most patients underwent an aortic valve replacement (43.2%, 82/190), followed by root replacement (18.4%, 35/190). The most common diagnosis was valvular heart disease (44.3%, 85/191), with aortic valve insufficiency (30.4%, 58/191) occurring more frequently than aortic valve stenosis (14.1%, 27/191) (Table 2). A bicuspid valve was present in 31.8% (41/129) of patients, and connective tissue disease was diagnosed in 3.6% (7/192). Among the seven patients with documented connective tissue disorders, five had Marfan syndrome, one had Ehlers–Danlos syndrome, and in one patient, a connective tissue disorder had histopathological confirmation; however, no specific diagnosis was documented in the medical records. Since the analysis spanned over two decades and many patients did not have their index surgery performed at our clinic, information regarding root size during the index operation and the reasons why root replacement was not performed at that time is missing. Moreover, 18.4% (35/190) of patients who underwent aortic root reoperation had previously undergone aortic root replacement (“true redo”) as their index surgery.

### 3.3. Data on Reoperation

The mean time between the index operation and reoperation was 8.61 (3.01–16.05) years.

The predominant indication for undergoing reoperation was the dilatation of the aortic root (62%, 119/192), followed by endocarditis (25%, 48/192), dissection (17.7%, 34/192), and false aneurysm formation (5.7%, 11/192). The most frequently performed procedure at reoperation was the Bentall or valve-sparing procedure (93.7%, 180/192). A total of 6.3% (12/192) of the patients underwent a variety of individualized root procedures, including modified Yacoub technique, root enlargements, aortic annulus reconstruction, non-coronary sinus reconstructions using patches, and repair of the aorto-mitral continuity (Commando procedure). Almost half of the patients required surgery in an urgent or salvage setting (Table 3).

### 3.4. Periprocedural Data

Mean operation duration was 370 (300–452) min, with a CPB time of 194 (149–238) min and an aortic cross-clamp time of 129 (103–157) min. Patients stayed in the intensive care unit (ICU) for approximately 4 (2–8) days, and the mean hospital stay duration was 14 (10–20) days (Table 4).

### 3.5. Intrahospital Postoperative Events

A close analysis was conducted on postoperative events during the ICU stay. Transfusion due to bleeding, according to BARC criteria, was required in 16.2% (31/191) of patients. Reintervention due to tamponade or hemothorax was necessary in 15.6% (30/192).

Moreover, the need for temporary mechanical circulatory support was observed in 7.3% (14/191), and neurovascular complications occurred in 13.5% (26/192) of the study population.

Postoperative complications are listed in Table 5.

### 3.6. Mortality and Other Outcomes

**Figure 1:**
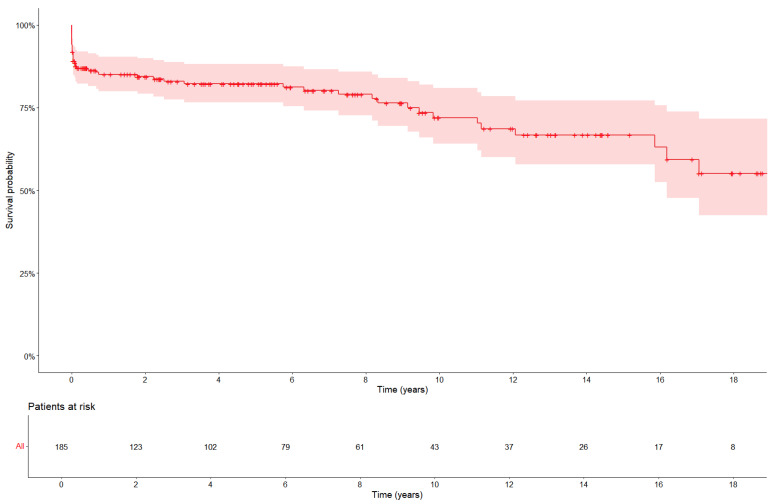
Kaplan–Meier curve for overall mortality after reoperation. The 30-day mortality was 13%.

The main aim of the current study was to assess all-cause mortality. After reoperation, 51 out of 192 patients died, resulting in an all-cause mortality of 26.6%. In total, 4.2% (8/192) of the patients died during the operation itself, 8.3% (16/192) of the patients died postoperatively but in-hospital, and 14.1% (27/192) of patients died after discharge. The 30-day mortality was 13% (25/192). Overall survival was 84%, 81%, and 71% at 1, 5, and 10 years, respectively (Figure 1). The main causes of death included cardiovascular causes (49%, 25/51), bleeding (15.7%, 8/51), and non-cardiac causes such as sepsis (7.8%, 4/51). The cause of death was unknown in 14 patients.

Cardiovascular mortality was defined as death due to myocardial infarction, sudden cardiac death, heart failure, cardiogenic shock, or stroke. Among cardiovascular deaths, cardiogenic shock due to pump failure occurred in 72% (18/25), sudden death in 12% (3/25), stroke in 8% (2/25), and myocardial infarction in 8% (2/25) of patients (Table S1). Among the patients who died due to bleeding, five experienced intraoperative exsanguination, one patient died in the ICU on the first postoperative day due to massive bleeding, and two patients died after hospital discharge. Of these, one suffered a ruptured abdominal aortic aneurysm, and the other died six months postoperatively from bleeding of unknown origin. The latter patient had a mechanical valve and was receiving anticoagulation therapy.

The additional aims of this study were to assess the incidence of cardiac re-reoperation, bleeding, new aortic dissection or rupture, infective endocarditis, readmission due to cardiac causes, cardiovascular and cerebral complications, pacemaker implantation, and tMCS implantation.

A cardiac re-reoperation in the follow-up time after entering this study was performed in 12.5% (24/192) of patients, with a median time to re-reoperation of 2.1 (0.36–9.90) years. Indications for re-reoperations included endocarditis (n = 9), dissection of the arch or the descending aorta (n = 9), hemodynamically significant paravalvular leaks (n = 2), cardiac decompensation due to severe valvular insufficiency of the aortic and mitral valve (n = 1), and bleeding (n = 3). A readmission due to a cardiac cause was necessary in 35.5% (67/190) of patients. After discharge, 6.8% (13/191) developed infective endocarditis, and nine of them (4.7%) required a new operation. Stroke after discharge was diagnosed in 8.9% (10/192). The principal and secondary outcomes are detailed in Table S1.

### 3.7. Risk Factor Analysis

We conducted a univariate analysis using Cox proportional hazards models to identify risk factors for mortality spanning from the time of reoperation through 20 years of the follow-up time. Several variables were found to be significant predictors of mortality, including gender (HR = 0.54, *p* = 0.04), age (HR = 1.06, *p* = 0.01), concomitant procedures at reoperation (HR = 2.98, *p* < 0.01), and combined aortic root and arch procedures at reoperation (HR = 2.23, *p* = 0.02). The results of the univariate regression analysis are shown in detail in Table S2.

To avoid overfitting nested Cox proportional hazards models, variables that were statistically significant in the univariate analysis were tested in multivariate regression analysis. In a second step, multivariate adjustments for all confounders with statistical significance in the univariate regression analysis were tested. Age, EuroSCORE II, prolonged CPB time, need for tMCS, bleeding complications, and concomitant procedures at reoperation were associated with a worse prognosis (Table 6 and Table 7).

### 3.8. Subgroup Survival Analysis

**Figure 2:**
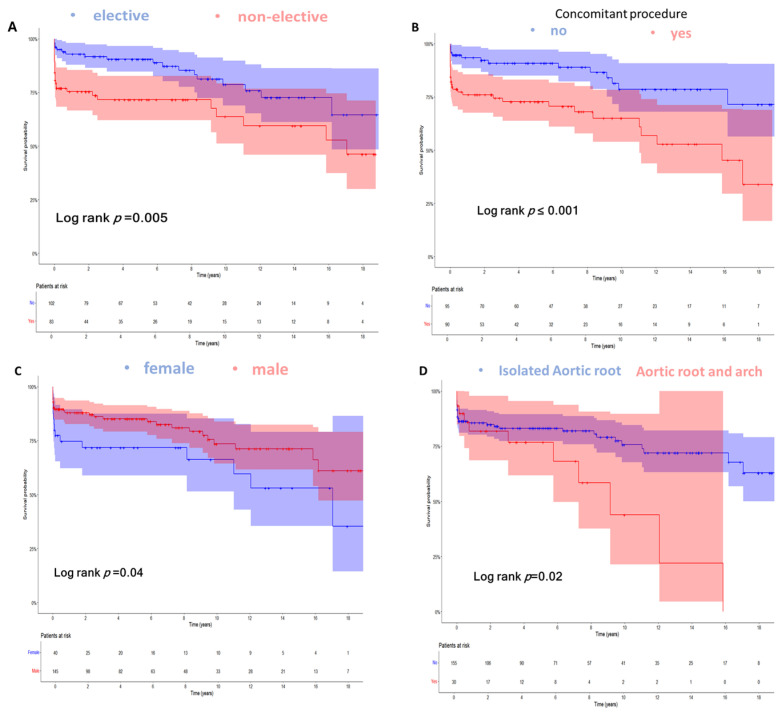
Kaplan–Meier curves demonstrating the overall survival over 18 years stratified for: (**A**) Electiveness of reoperation (mean survival for elective reoperation 17.5 (95% CI: 15.5–19.6) vs. 12.2 (95% CI: 10.1–14.2) years for non-elective reoperations; Log rank *p* < 0.01). (**B**) Patients not receiving concomitant procedure in addition to aortic root reoperation vs. patients receiving concomitant procedure at reoperation (mean survival 18.4 (95% CI: 16.4–20.3) vs. 11.6 (95% CI: 9.7–13.5) years, respectively; Log rank *p* < 0.01). (**C**) Gender (mean survival for male gender 16.7 (95% CI: 14.9–18.5) vs. 12.4 (95% CI: 9.3–15.5) years for female gender; Log rank *p* = 0.04). (**D**) Patients not receiving the combined procedure at the aortic arch at reoperation vs. patients receiving the combined procedure at the aortic arch at reoperation (mean survival 16.8 (95% CI: 15.1–18.5) vs. 8.9 (95% CI: 6–11.8) years, respectively; Log rank *p* = 0.02). Overall survival was defined as the time in years from reoperation on the aortic root until the end of follow-up (September 2023) or the incidence of death.

We conducted an additional subgroup analysis to explore differences in overall survival across the following subgroups, which showed a significant difference in the previous analysis: (a) electiveness of reoperation, (b) receipt of concomitant procedures, (c) gender, and (d) combined procedures at the aortic arch. Patients undergoing an elective operation had significantly better survival of 17.5 years (95% CI: 15.5–19.6), compared to non-elective patients at 12.2 years (95% CI: 10.1–14.2) (Log rank *p* < 0.01). Also, the need for concomitant procedures in addition to aortic root reoperation was associated with a significantly impaired survival. Furthermore, female gender and combined procedures involving the aortic arch resulted in poorer survival (Figure 2).

### 3.9. Sensitivity Analysis

To better assess long-term outcomes, we performed a sensitivity analysis in which we excluded patients with less than 30 days between the index operation and reoperation, as this is associated with increased mortality. Conduction of sensitivity analysis confirmed the significance of electiveness at the time of the reoperation and the negative influence of concomitant procedures during the reoperation.

### 3.10. ROC Curve and Survival Analysis

**Figure 3:**
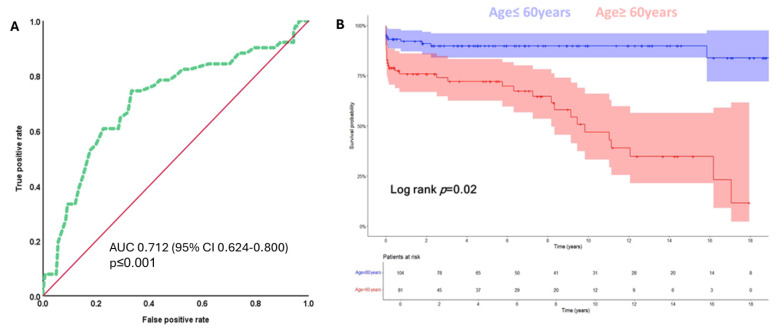
(**A**) ROC curve for age in years for the prediction of mortality during follow-up. The area under the curve (AUC) indicates prognostic value for age for predicting death in patients undergoing reoperation on the aortic root (AUC = 0.712; 95% CI: 0.624–0.800, *p* < 0.01). The cut-off value for age in years chosen with the best Youden index for the prediction of mortality was 60 years (sensitivity 74.5%, specificity 66.7%). (**B**) Stratified Kaplan–Meier survival analysis for the overall survival of patients who are ≤ 60 years old is significantly better as compared to those who are older than 60 years at the time of reoperation (mean survival 19.9 (95% CI: 18.4–21.4) vs. 9.5 (95% CI: 7.7–11.3) years, respectively; Log rank test *p* = 0.02). Overall survival was defined as the time in years from reoperation on the aortic root until the end of follow-up (September 2023) or the incidence of death.

Based on the optimal Youden index for predicting mortality, ROC analysis yielded a cut-off value of 60 years with a sensitivity of 74.5% and specificity of 66.7%. Kaplan–Meier survival analysis, stratified by the age cut-off of 60 years, demonstrated significantly poorer survival among older patients (Figure 3).

## 4. Discussion

Reoperation of the aortic root is one of the most demanding procedures in cardiac surgery due to the technical complexity of the procedure in a high-risk patient population.

Long-term data of patients undergoing a reoperation of the aortic root are sparse, the results for first vs. second sternotomy are controversial, and elective and emergency surgeries are often not analyzed separately [7,9,10]. The aim was to analyze the long-term results after reoperation on the aortic root in a high-volume center and to identify risk factors for mortality in patients who already had previous surgery on the aorta or the aortic valve.

The main finding of our study was that mortality was primarily driven by operative and in-hospital mortality (12.4% of the study population), with a favorable prognosis after discharge (approximately 2% mortality rate per year, evenly distributed) and an acceptable risk of cardiac re-reoperation (12.5%). Additionally, factors such as age, female gender, non-elective surgery, combined aortic root and aortic arch procedures, and concomitant procedures during reoperation were associated with lower survival.

The increasing number of cardiac reoperations can be attributed to more complex patients and better survival after cardiac surgery. Despite well-performed surgery at the index procedure and secondary prevention by strict adherence to guidelines, degeneration of prosthetic valves, dilatation of the aortic root, dissections, and endocarditis may occur [1,2]. In cases of isolated root dilatation, it is worth asking whether the aortic root should have been replaced during the index operation, particularly in patients with bicuspid valves or connective tissue disease. Unfortunately, most index procedures in our cohort were performed externally, and root measurements were not available.

### 4.1. Risk Factors for Adverse Outcomes

In our study we identified several risk factors associated with increased mortality after aortic root reoperation. Advanced age, diabetes, and higher EuroSCORE II were found to be significant predictors of poor prognosis. Silva et al. reported that patients needing a reoperation on the ascending aorta and aortic root had a high-risk profile, with higher EuroSCORE II, but despite that, they showed reasonable outcomes in highly experienced centers (1-year survival 77.9%) [2]. However, in contrast to our study, Silva et al. included patients with various index operations, which makes comparison difficult [2]. Esaki et al. showed in their publication that patients with chronic lung disease and a history of myocardial infarction who underwent reoperation on the aortic root had a significantly higher mortality rate [11]. To improve surgical outcomes, careful patient selection is essential [12]. Our study showed that patients with comorbidities often have protracted postoperative courses, including longer stays in the ICU, increased need for mechanical circulatory support, and higher rates of re-exploration. Using the ROC curve, age was found to be a significant predictor of mortality. The analysis showed that patients over 60 years had a significantly worse prognosis. Our study is the first study using this analysis to set a cut-off value, helping with preoperative patient risk stratification.

### 4.2. The Influence of Urgency and Combined Cardiac Surgery

The urgency of a reoperation has a significant impact on outcomes. Therefore, patients who underwent emergency and elective surgery were analyzed separately. Patients undergoing emergency or urgent surgeries had significantly impaired survival rates compared to elective patients (elective 17.5 years (95% CI: 15.5–19.6) vs. non-elective 12.2 years (95% CI: 10.1–14.2); Log rank *p* < 0.01). These findings match the findings of Levine et al. and Feng et al., who showed that patients with endocarditis and type A aortic dissection had worse outcomes than elective reoperation of the aortic root, respectively [13,14]. In contrast to our study, Deng et al. showed that the prognosis of reoperation on the aortic root is not influenced by the indication. According to Deng et al., this finding could be attributed to improvements in imaging technology, the increased use of biological heart valves, and the rising age of patients undergoing surgery. However, this was a small heterogeneous collective that expanded over a long period of time [3]. In addition, the complexity of the reintervention itself was a significant outcome driver. Patients who required combined procedures (e.g., arch replacement, concomitant CABG, mitral valve surgery) had a significantly higher mortality rate (survival: 16.8 (95% CI: 15.1–18.5) vs. 8.9 (95% CI: 6–11.8) years, *p* = 0.02) [15], due to very long operating and aortic clamping times that lead to hemodynamic instability [10,15,16,17,18,19].

Although our study focused on open surgical reoperations, which remain the standard of care in complex aortic root pathology, particularly in the context of prior surgery, endocarditis, dissection, or coronary button reimplantation. Development of new techniques such as hybrid procedures, including also sutureless valves, can shorten the long operating times in selected patients and thus could improve the outcome in elderly and more vulnerable patients [13,20,21]. For instance, in an elderly patient needing reoperation on the aortic root along with bypass surgery or mitral valve surgery, the aortic root could be replaced initially, while the coronary arteries or other valves could be treated transcatheter at a later time. Even though minimally invasive and hybrid approaches hold promise for selected high-risk patients, their use in reoperations on the aortic root is still limited due to anatomical constraints and the technical complexity of reimplanting coronary arteries. The sensitivity analysis enabled us to substantiate our results, particularly regarding the importance of elective versus non-elective operations and the effects of concomitant procedures.

### 4.3. Endocarditis as a Critical Factor

Endocarditis was a common diagnosis for reoperation in our study population and was associated with a significantly worse prognosis in the univariate analysis (HR = 2.00, *p* = 0.03). Patients with endocarditis had a higher rate of postoperative complications, including bleeding, need for circulatory support, and longer ICU stays. The presence of endocarditis not only complicates the surgical procedure but also increases the risk of postoperative sepsis and reinfection and is associated with worse neurological outcomes [10,14,22]. The use of hemoadsorption during CPB, particularly in patients with endocarditis and an impaired inflammatory response, could improve outcomes [23].

### 4.4. Aortic Root Reoperation After Initial Aortic Root Surgery (True Aortic Root Redo)

Our study found that patients with previous root replacement surgery, particularly those with a history of Bentall procedure, faced increased risks when undergoing root replacement as reoperation. Major complications observed in our study population after aortic root reoperation included bleeding requiring re-intervention (16.2%), need for temporary mechanical circulatory support (7.3%), neurovascular events (13.5%), and reoperation mortality (13% at 30 days). Patel et al. further investigated the outcomes of root replacement reoperations and distinguished between true root reoperation (at the same anatomical site) and reoperation after other aortic surgeries. Their results showed that true redo carries higher surgical risks due to extensive adhesions, distorted anatomy, and the need for re-mobilization of the coronary arteries. Nevertheless, his study showed good clinical results after a true redo-root operation [24]. Therefore, in order to achieve optimal results, the individualization of the surgical strategy, including the selection of the most suitable prosthesis and addressing the entire diseased segment of the aorta when indicated, could help reduce future reoperation rates. However, these decisions must be balanced with patient-specific factors such as age, comorbidities, and surgical risk [6].

### 4.5. Gender Differences in Outcomes

An interesting finding from our study was the association between female gender and lower overall survival after aortic root reoperation survival in years: male 16.7 (95% CI: 14.9–18.5) vs. female 12.4 (95% CI: 9.3–15.5) (*p* = 0.04). This was also a finding in the study by Bechtel et al. [25]. Women who undergo surgery tend to be older, with comorbidities and frailty. Additionally, according to Al-Ebrahim et al., the delayed presentation of women compared to men may play a significant role [26].

### 4.6. Long-Term Outcomes and Survival Analysis

Although our study showed that short-term mortality after aortic root reoperation was high (30-day mortality 13%), patients who survived the first postoperative period had good long-term outcomes. Overall survival rates at 1, 5, and 10 years were 84%, 81%, and 71%, respectively, being in line with data from other studies [1,10].

However, the survival benefit decreases significantly in patients over 60 years of age, as shown by our sensitivity analysis and ROC curve evaluation. Older patients had a significantly worse prognosis, with a sharp decline in survival rate at 10 years. These results suggest that age should be a central consideration in preoperative risk stratification and decision-making, potentially leading to more conservative management in selecting older patients.

### 4.7. Study Limitations

The main limitation of this study is its retrospective design. Despite rigorous data collection, reliance on medical records and follow-up may have led to underreporting of complications or events.

In addition, our study was conducted at a highly specialized center with high patient volume and extensive experience in aortic surgery and postoperative intensive care management. Multicenter studies with larger patient populations are needed to substantiate our findings.

Finally, our study did not explore the impact of specific surgical techniques on outcomes. Future research should aim to analyze the role of innovative surgical approaches such as hybrid procedures to achieve better outcomes in high-risk patients.

## 5. Conclusions

In this study, mortality was primarily driven by operative and intrahospital mortality. After discharge, patients showed a good prognosis and an acceptable risk of cardiac re-reoperation. Further, age appeared to be a significant outcome driver. A cut-off value of 60 years is a predictor of mortality. Moreover, female gender, non-elective surgery, combined aortic root and aortic arch procedures, and receiving concomitant procedures at reoperations are associated with decreased overall survival. Finally, higher EuroSCORE II, prolonged cardiopulmonary bypass time, bleeding complications requiring tCMS, and concomitant procedures at reoperations are predictors of mortality independently of multiple confounders.

## Figures and Tables

**Table 1:** Preoperative characteristics of the study participants at the time or reoperation on the aortic root.

Preoperative Characteristics	
Male n (%)	149/192 (77.6%)
Age (years)	57 ± 13
BMI (kg/m^2^)	27.24 ± 5.43
Arterial hypertension	67/159 (42.1%)
Diabetes mellitus	13/188 (7%)
Dyslipidemia	55/187 (29.4%)
History of CAD	44/182 (42.2%)
Smoking history	59/180 (32.8%)
History of myocardial infarction	7/188 (3.7%)
History of neurovascular event	24/188 (12.8%)
Chronic lung disease	21/132 (15.9%)
Peripheral artery disease	4/188 (2.1%)
Chronic kidney disease	20/185 (10.8%)
Creatinine (mg/dL)	1.07 ± 0.62
Chronic liver disease	8/186 (4.3%)
EuroSCORE II (%)	5.08 (2.38–10.09)
LVEF (%)	60 (54–61)
LVEF	
Normal (≥50%)	110/137 (80.3%)
Mildly reduced (40–49%)	18/137 (9.4%)
Moderately reduced (30–39%)	6/137 (4.4%)
Severely reduced (<30%)	3/137 (2.2%)
NYHA	
I	12/161 (7.5%)
II	38/161 (23.6%)
III	64/161 (39.8%)
IV	47/161 (29.2%)
Bicuspid aortic valve	41/129 (31.8%)
Connective tissue disease	7/192 (3.6%)
Number of sternotomies before reoperation	
1	149/191 (78%)
2	37/191 (19.4%)
3	5/191 (2.6%)

BMI: body mass index; CAD: coronary artery disease; LVEF: left ventricular ejection fraction; NYHA: New York Heart Association.

**Table 2:** Detailed data regarding performed procedures, diagnosis/indication and type of prosthetic valve at the time of index operation of the study participants.

Index Operation	
Procedure at index operation	
Aortic valve replacement	82/190 (43.2%)
Supracoronary ascending aortic replacement	21/190 (11.1%)
Aortic valve and aorta ascendens replacement	25/190 (12.1%)
Root replacement	35/190 (18.4%)
Arch replacement and descending thoracic aorta stenting	4/190 (2.1%)
Resection of coarctation of the aorta	12/190 (6.3%)
Combination	13/190 (6.8%)
Concomitant procedures on previous operation	23/190 (12.1%)
Diagnosis at index operation	
Dilatation	15/191 (7.9%)
Rupture/Dissection	37/191 (19.3%)
Endocarditis	15/191 (7.8%)
Valvular disease	85/191 (44.3%)
Valvular disease and dilatation	37/191 (19.3%)
Other	2/191 (1%)
Prosthetic valve type on aortic position at index operation	
Biological	59/187 (31.6%)
Mechanical	72/187 (38.5%)
Aortic valve neocuspidization (Ozaki procedure)	2/187 (1.1%)
Reconstruction	9/187 (4.8%)

**Table 3:** Detailed data regarding diagnosis/indication leading to reoperation, procedure on aortic root, concomitant procedures performed and the state of urgency at the time of reoperation.

Reoperation Data	
Diagnosis at reoperation	
Dilatation	119/192 (62%)
Rupture/False aneurysm	11/192 (5.7%)
Dissection	34/192 (17.7%)
Endocarditis	48/192 (25%)
Procedure on aortic root at reoperation	
Bentall	170/192 (88.5%)
David	6/192 (3.1%)
Yacoub	4/192 (2.1%)
Other	12/192 (6.3%)
Combined procedure on aortic root and arch at reoperation	32/192 (16.7%)
Descending thoracic aortic stent–graft placement	6/191 (3.14%)
Prosthetic valve type in aortic position after reoperation	
Biological	95/192 (49.5%)
Mechanical	79/192 (41.1%)
Concomitant procedures at reoperation	
Operation on aorta	30/192 (15.6%)
CABG	28/192 (14.6%)
Mitral valve	9/192 (4.7%)
Tricuspid valve	3/192 (1.6%)
Other	7/192 (3.6%)
Combination	13/192 (6.8%)
Commando procedure	5/192 (2.6%)
Urgency of reoperation	
Elective	105/192 (54.6%)
Urgent	51/192 (26.6%)
Emergency	28/192 (14.6%)
Salvage	8/192 (4.2%)

CABG: coronary artery bypass graft.

**Table 4:** Periprocedural data and length of ventilation time, ICU stay and hospital stay after reoperation on the aortic root.

Periprocedural Data	
Duration (min)	370 (300–452)
Cardiopulmonary Bypass Time (min)	194 (149–238)
Aorta clamp time (min)	129 (103–157)
Reperfusion time (min)	39 (24–59)
Single ischemia max (min)	124 (92–154)
Second time on heart–lung machine	5/192 (2.6%)
Second time clamping of aorta	5/192 (2.6%)
Ventilation time (days)	1 (1–3)
ICU stay (days)	4 (2–8)
Hospital stay (days)	14 (10–20)

ICU: intensive care unit.

**Table 5:** Postoperative intrahospital complications following reoperation on the aortic root.

Postoperative Complications (Intrahospital)	
Gastrointestinal bleeding requiring transfusion	4/191 (2.1%)
Neurovascular complication	26/192 (13.5%)
Delirium	13/190 (6.8%)
Requiring coronary intervention	2/192 (1%)
Bleeding requiring transfusion or intervention	31/191 (16.2%)
Cardiac tamponade or hemothorax	30/192 (15.6%)
Requiring temporary MCS	14/191 (7.3%)
Requiring a permanent pacemaker	27/192 (14.1%)

**Table 6:** Nested multivariate Cox Proportional Hazards Models on the incidence of death. Each model examines clinically related confounders. Model 1 investigates factors related to medical history. Model 2 adjusted for variable of preoperative characteristics. Model 3 evaluates operative times and postoperative data. Adjustment for age and gender was performed in all models.

Multivariate Cox Proportional Hazards Models Regarding the Incidence of Death			
Variable	Hazard Ratio	95% Confidence Interval	*p*
**Model 1: Medical history**			
Male gender *	1.01	0.45–2.26	0.98
Age ≥ 60 years	2.20	0.80–6.00	0.10
Concomitant procedure at index operation *	1.10	0.45–2.68	0.804
Biological prosthesis at index operation	1.23	0.51–2.98	0.65
EuroSCORE II (%)	1.46	1.17–1.81	<0.01
Coronary artery disease *	1.47	0.68–3.19	0.33
**Model 2: Preoperative variables**			
Male gender *	0.79	0.38–1.61	0.51
Age ≥ 60 years	3.98	1.81–8.76	<0.01
Reoperation diagnosis: dilatation	1.28	0.53–3.09	0.59
Reoperation diagnosis: false aneurysm	1.92	0.75–4.88	0.17
Reoperation diagnosis: endocarditis	1.60	0.73–3.49	0.24
Biological prosthesis at reoperation	1.61	0.74–3.48	0.23
Non-electiveness of operation	1.63	0.70–3.75	0.25
Concomitant procedure at reoperation	2.53	1.32–4.85	0.01
**Model 3: Operative times and postoperative complications**			
Male gender *	0.66	0.34–1.28	0.22
Age ≥ 60 years	4.21	2.07–8.54	<0.01
Cardiopulmonary bypass time (minutes)	1.01	1.01–1.02	<0.01
Aorta clamp time (minutes)	0.99	0.99–1.00	0.16
ICU stay (days)	0.96	0.90–1.03	0.23
Ventilation (days)	1.05	0.99–1.12	0.11
Bleeding after reoperation *	6.11	3.02–12.37	<0.01
Temporary MCS at reoperation *	4.86	1.69–13.95	<0.01

* Reference category: absence of characteristic. ICU: intensive care unit; MCS: mechanical circulatory support.

**Table 7:** Multivariate Cox proportional hazards model regarding the incidence of death.

Variable	Hazard Ratio	95% Confidence Interval	*p*
Age > 60 years	3.42	1.13–10.42	0.03
EuroSCORE II (%)	1.35	1.02–1.75	0.03
Reoperation diagnosis: endocarditis	4.13	1.21–14.07	0.02
Cardiopulmonary bypass time (minutes)	1.01	1.00–1.02	0.05
Bleeding after reoperation *	9.00	3.49–23.22	≤0.001
Temporary MCS at reoperation *	4.33	1.24–15.15	0.02

* Reference category: absence of characteristic Additionally adjusted for and found not significant: male gender, concomitant procedure at index operation, biological prosthesis at index operation, coronary artery disease, reoperation diagnosis: dilatation, reoperation diagnosis: false aneurysm, non-electiveness of operation, concomitant procedure at reoperation, aorta clamp time (minutes), ICU stay (in days), and ventilation (in days). ICU: intensive care unit; MCS: mechanical circulatory support.

## Data Availability

The data that support the findings of this study are available from the corresponding author upon reasonable request.

## Supplementary Materials

The following supporting information can be downloaded at: https://www.mdpi.com/article/10.3390/jcm14113727/s1. Table S1: Mortality and other outcomes; Table S2: Univariate analysis for the incidence of death.
