# Supplementary material for: Long-Term Outcomes and Risk Factors of Mortality After Reoperation on the Aortic Root: A Single-Center 20-Year Experience
Source: J Clin Med. 2025 May 26;14(11):3727. doi: 10.3390/jcm14113727 (PMC12155750; doi:10.3390/jcm14113727)
Supplement: Supplementary file 1 [file jcm-14-03727-s001.zip › jcm-3640477-supplementary.pdf]

Supplementary Material

Table S1

**Outcomes**	
**Mortality**	51/192 (26.6%)
**Cause of Death**	
Cardiovascular	25/192 (13.0 %)
Bleeding	8/192 (4.2%)
Non cardiac	4/192 (2.0%)
Unknown	14/192 (7.3%)
**Time of Death**	
Intraoperative	8/192 (4.2%)
Intrahospital	16/192 (8.3%)
Post-Discharge	27/192 (14.1%)
**Cardiac re-reoperation**	24/192 (12.5%)
**Other clinical outcomes after discharge**	
Dissection/Rupture/Aneurysma	9/191 (4.7%)
Pacemaker Implantation	5/192 (2.6%)
Coronary intervention	2/192 (1%)
Stroke	10/192 (8.9%)
Bleeding requiring transfusion	6/192 (3.1%)
Endocarditis	13/191 (6.8%)
Readmission due to cardiac cause	67/190 (35.5%)

Table S2. Univariate Analysis for the incidence of death.

Variable	HR	95% CI	P Value
**Preoperative characteristics**			
Male	0.54	0.29-0.99	**0.04**
Age (years)	1.06	1.03-1.09	**<0.01**
BMI (kg/m²)	1.02	0.95-1.010	0.57
Arterial hypertension	0.88	0.48-1.74	0.72
Diabetes mellitus	5.48	2.36-12.64	**<0.01**
Dyslipidemia	0.91	0.46-1.82	0.79
History of coronary artery disease	1.92	0.99-3.74	0.06
Smoking history	1.05	0.53-2.08	0.89
History of myocardial infarction	0.99	0.14-7.25	0.99
History of neurovascular event	1.89	0.87-4.10	0.11
Chronic lung disease	0.73	0.22-2.40	0.60
Peripheral artery disease	12.55	3.65-43.14	**<0.01**
Chronic kidney disease	2.10	0.73-6.00	0.17
Creatinine (mg/dl)	1.55	1.13-2.14	**0.01**
Chronic liver disease	2.60	0.79-8.48	0.11
EuroSCORE II (%)	1.04	1.02-1.06	**<0.01**
LVEF (%)	0.97	0.93-0.99	**0.03**
LVEF ≤moderate reduced (≤39%)	2.35	1.11-4.95	**0.02**
NYHA ≥III	1.43	0.65-3.15	0.38
Bicuspid aortic valve	0.67	0.29-1.58	0.36

Connective tissue disease	0.05	0.01-5.40	0.39
≥2 sternotomies before reoperation	1.02	0.51-2.06	0.95
Procedure on previous operation			
Aortic valve procedure	1.07	0.59-1.92	0.83
Supracoronary ascending aorta replacement	1.21	0.51-2.87	0.66
Aortic valve and aorta ascendens replacement	0.34	0.08-1.42	0.14
Root replacement	1.13	0.55-2.36	0.73
Arch replacement and/or aorta descendens stenting	1.61	0.39-6.70	0.51
Resection of coarctation of aorta	0.56	0.14-2.31	0.42
Combination	1.69	0.60-4.76	0.32
Concomitant procedures at index operation	2.00	02.92-4.33	0.08
Diagnosis at index operation			
Dilatation	0.79	0.25-2.57	0.70
Rupture/Dissection	1.38	0.70-2.73	0.35
Endocarditis	1.16	0.41-3.26	0.78
Valvular disease	1.04	0.58-1.86	0.91
Valvular disease and dilatation	0.73	0.31-1.73	0.47
Other	0.048	0.01-84.6	0.54
Prosthetic valve type at index operation			0.47
Biological	2.91	1.51-5.60	**<0.01**
Mechanical	0.65	0.35-1.27	0.19
Aortic valve neocuspidization (Ozaki procedure)	0.05	0.01-121.3	0.69
Reconstruction	0.39	0.05-2.86	0.36
Valvuloplasty	0.05	0.01-53.88	0.61
Diagnosis at reoperation			
Dilatation	0.55	0.31-0.99	**0.04**
Rupture/Endoleak	2.55	1.08-6.05	**0.03**
Dissection	1.08	0.50-2.32	0.84
Endocarditis	2.00	1.08-3.69	**0.03**
Procedure on aortic root at reoperation			
Bentall	0.68	0.28-1.59	0.36
David	1.29	0.30-5.11	0.78
Yacoub	2.32	0.55-9.56	0.25
Other	1.42	0.34-5.92	0.63
Combined procedure on aortic root and arch at reoperation	2.23	1.11-4.46	**0.02**
Procedure on descending aorta	4.29	1.52-12.04	**0.02**
Prosthetic valve type in aortic position after reoperation			
Biological	3.25	1.70-6.23	**<0.01**
Mechanical	0.22	0.10-0.46	**<0.01**
Concomitant procedure at reoperation	2.98	1.58-5.62	**<0.01**
Concomitant procedures at reoperation			
Operation on PROXIMAL aorta	1.63	0.81-.3.30	0.18
CABG	2.48	1.26-4.91	**0.01**
Operation at mitral or tricuspid valve	2.27	1.06-4.88	**0.04**
Other or combined procedures	0.57	0.14-2.34	0.43
Urgency of reoperation			

Elective	0.43	0.24-0.79	**0.01**
Urgent***	1,36	0.72-2.56	0.35
Emergency***	1.18	0.53-2.65	0.69
Salvage***	16.42	6.32-42.69	**<0.01**
Operative Data			
Duration (min)	1.00	0.99-1.01	0.70
Cardiopulmonary Bypass Time (min)	1.01	1.01-1.02	**<0.01**
Aorta clamp time (min)	1.01	1.01-1.02	**0.01**
Reperfusion time (min)	1.02	1.01-1.02	**<0.01**
Single ischemia max (min)	1.01	1.01-1.02	**0.01**
Second time on heart-lung machine	1.01	0.98-1.01	0.11
Second time clamping of aorta	1.01	1.01-1.03	0.04
Ventilation time (days)	1.04	1.01-1.05	**<0.01**
ICU stay (days)	1.03	1.01-1.05	**<0.01**
Hospital stay (days)	1.01	0.99-1.03	0.15
Postoperative complications			
Gastrointestinal bleeding requiring transfusion or intervention	0.31	0.08-1.30	0.11
Neurovascular complications	1.55	0.75-3.22	0.24
Delirium	0.69	0.17-2.87	0.61
Percutaneous coronary intervention	0.05	0.01-61.16	0.67
Bleeding requiring transfusion	4.82	2.65-8.75	**<0.01**
Tamponade or Haematothorax	2.90	1.53-5.46	**<0.01**
Requiring MCS	15.53	7.46-32.28	**<0.01**
Requiring pacemaker	0.53	0.16-1.70	0.28

*** Reference category all other states of urgency for reoperation (including elective).
